# Narcolepsy and H1N1 influenza immunology a decade later: What have we learned?

**DOI:** 10.3389/fimmu.2022.902840

**Published:** 2022-10-12

**Authors:** Sofia M. Buonocore, Robbert G. van der Most

**Affiliations:** GSK, Rixensart, Belgium

**Keywords:** narcolepsy, H1N1 influenza, vaccine, hypocretin, Pandemrix, CD4+ T cells

## Abstract

In the wake of the A/California/7/2009 H1N1 influenza pandemic vaccination campaigns in 2009-2010, an increased incidence of the chronic sleep-wake disorder narcolepsy was detected in children and adolescents in several European countries. Over the last decade, in-depth epidemiological and immunological studies have been conducted to investigate this association, which have advanced our understanding of the events underpinning the observed risk. Narcolepsy with cataplexy (defined as type-1 narcolepsy, NT1) is characterized by an irreversible and chronic deficiency of hypocretin peptides in the hypothalamus. The multifactorial etiology is thought to include genetic predisposition, head trauma, environmental triggers, and/or infections (including influenza virus infections), and an increased risk was observed following administration of the A/California/7/2009 H1N1 vaccine *Pandemrix* (GSK). An autoimmune origin of NT1 is broadly assumed. This is based on its strong association with a predisposing allele (the human leucocyte antigen DQB1*0602) carried by the large majority of NT1 patients, and on links with other immune-related genetic markers affecting the risk of NT1. Presently, hypotheses on the underlying potential immunological mechanisms center on molecular mimicry between hypocretin and peptides within the A/California/7/2009 H1N1 virus antigen. This molecular mimicry may instigate a cross-reactive autoimmune response targeting hypocretin-producing neurons. Local CD4^+^ T-cell responses recognizing peptides from hypocretin are thought to play a central role in the response. In this model, cross-reactive DQB1*0602-restricted T cells from the periphery would be activated to cross the blood-brain barrier by rare, and possibly pathogen-instigated, inflammatory processes in the brain. Current hypotheses suggest that activation and expansion of cross-reactive T-cells by H1N1/09 influenza infection could have been amplified following the administration of the adjuvanted vaccine, giving rise to a “two-hit” hypothesis. The collective *in silico*, *in vitro*, and preclinical *in vivo* data from recent and ongoing research have progressively refined the hypothetical model of sequential immunological events, and filled multiple knowledge gaps. Though no definitive conclusions can be drawn, the mechanistical model plausibly explains the increased risk of NT1 observed following the 2009-2010 H1N1 pandemic and subsequent vaccination campaign, as outlined in this review.

## Introduction

In the aftermath of the A/California/7/2009 H1N1 (A/H1N1pdm09) influenza pandemic in 2009-2010, an increased incidence of the sleep-wake disorder narcolepsy was observed in several European countries, mostly in children and adolescents who received *Pandemrix* (GSK) (1–5). This AS03-adjuvanted (6, 7) inactivated split-virion A/H1N1/pdm09 vaccine was manufactured in Germany and predominantly used in Europe during the pandemic. Given the influenza strain’s persistent circulation, vaccination continued in the UK on a small scale (~148,000 individuals) through the end of the 2010-2011 season, in situations when seasonal vaccine supply was limited (8). The narcolepsy vaccine safety signal derived from these rare events triggered intense research efforts to expand the epidemiological knowledge, and to generate evidence informing a hypothetical pathogenesis mechanism, which centered on an autoimmune etiology (9, 10). The collective, multidisciplinary research has progressively enabled to refine and extend the initially posed hypotheses of the likely underlying mechanism. Though the evidence does not suffice to draw definitive conclusions, the emerging mechanistical model offers a plausible and coherent explanation for the association between *Pandemrix* vaccination and narcolepsy. This review synthesizes the research conducted over the last decade since the detection of the signal, with further mechanistic research still ongoing.

## Context

### AS03-adjuvanted pandemic influenza vaccines

Besides *Pandemrix*, a similar monovalent AS03-adjuvanted A/H1N1pdm09 vaccine (*Arepanrix*; GSK) was supplied during the pandemic. This vaccine was manufactured in Canada and distributed mostly in the Americas (Brazil, Canada, Mexico), Malaysia and Turkey. Of the estimated 90 million doses administered in total (*Pandemrix*/*Arepanrix*: 31/59 million), around 9.5 million were given to children (8). Single-dose regimens were generally administered, due to the high effectiveness of one dose of the vaccine, though in some countries a two-dose schedule was used to vaccinate immunocompromised adults and younger children (8).

The *Pandemrix* and *Arepanrix* vaccine antigens were derived from the NYMC X-179A reassortant influenza strain, where the haemagglutinin (HA), neuraminidase (NA) and polymerase basic 1 (PB1) originated from A/H1N1pdm09, and the remaining proteins from a 1934 A/H1N1 strain (PR8) (11). Clinical trials demonstrated that the two AS03-adjuvanted pandemic influenza vaccines share similar reactogenicity and immunogenicity profiles (12, 13).

AS03 is an oil-in-water Adjuvant System containing DL-α-tocopherol and squalene oil dispersed in water (6, 7). The safety profiles of the AS03-adjuvanted influenza vaccines observed in clinical trials conducted in adults and children were clinically acceptable (8). Reactogenicity was increased compared to non-adjuvanted influenza vaccines, but symptoms generally remained transient and, mild-to-moderate in intensity and mostly localized at the site of injection (8). In children, especially those younger than 3 years of age, increased incidence of fever was observed particularly after the second dose (13–15).

The adjuvant’s defined mode of action includes increased innate cytokine expression and antigen uptake by monocytes, as seen in mice (6), and activation of interferon pathways (measured through transcriptomics), as demonstrated in humans (16–19). Combined with different antigens, the innate immune activation translated into increased antigen-specific CD4^+^ (but not CD8^+^) T-cell responses, and quantitative/qualitative enhancement of specific antibody responses (11, 20–22). Another defined feature of AS03 activity, noted when used in combination with H5N1 antigen, is an epigenetic modification of innate cells, which augments the cells’ responsiveness upon subsequent viral exposure (17). A Phase 3 trial, comparing seasonal influenza vaccines with or without AS03 in older adults, although it missed its primary endpoint of preventing any influenza infection, has shown higher efficacy of the adjuvanted vaccine in preventing A/H3N2 infection and all-cause mortality or pneumonia (23). For (pre)pandemic H5N1 and A/H1N1pdm09 vaccines, AS03 enabled enhanced immunogenicity, antigen-sparing, persistence of immune response and cross-clade reactivity (20, 24).

Overall, the extensive experience gained with AS03-adjuvanted vaccines has yielded a substantial and comprehensive safety and immunogenicity database (8), and suggests that vaccines formulated with this adjuvant could be crucial to adequately address pandemic threats, not limited to influenza viruses (7).

### NT1 immunopathogenesis

Narcolepsy is a chronic neurological disorder that is primarily characterized by a reduced ability of the brain to control sleep-wake cycles and, in most individuals, cataplexy (an abrupt loss of muscle tone). When presenting in conjunction with cataplexy, the disorder is commonly referred to as type 1 narcolepsy (NT1). Though no cure is available, some symptoms, such as excessive daytime sleepiness and cataplexy, may be controlled by medication (25, 26). NT1 is furthermore associated with obesity and a range of psychosocial and psychiatric problems (27, 28).

Narcolepsy with cataplexy has a global prevalence of 25–50 per 100,000 individuals, with the onset of symptoms usually in childhood or adolescence (25). While it can have a post-traumatic etiology, a causal role of infections such as *Streptococcus* or influenza has also been suggested (25). NT1 is diagnosed by the Multiple Sleep Latency Test, as summarized by Bassetti et al. (25). Diagnosis can be supported by measuring levels of the neuropeptide hypocretin (HCRT) in the cerebrospinal fluid. For a more detailed overview of the NT1 clinical spectrum, pathophysiology, diagnosis, and treatment, we refer to the above-cited review by Bassetti et al. (25). Symptoms are likely caused by selective loss of the limited number [approximately 60,000 (29)] of hypothalamic neurons producing HCRT, resulting in a permanent and irreversible lack of HCRT signaling in the brain. Upon its secretion, the precursor hormone (prepro-HCRT) is processed into the HCRT1 and HCRT2 peptides which then undergo amidation, a post-translational modification essential for biological activity (30, 31). These peptides can bind to the HCRT receptors types 1 and 2 (HCRT-R1/R2), which are preferentially expressed in the central nervous system (CNS), by different cell types located throughout the brain—though most likely not by HCRT—producing neurons themselves (32). The resulting interactions are involved in the regulation of cognitive and physiological functions, including sleep/wake states.

The prevailing consensus is that NT1 has an autoimmune origin, similar to type 1 diabetes which is caused by autoimmunity to the secreted hormone prepro-insulin (33). A key feature of autoimmune diseases is a genetic association with specific human leukocyte antigen (HLA) alleles (34). The occurrence of NT1 is strongly linked to positivity for certain HLA class II (HLA-II) alleles. HLA-II molecules are involved in immune system regulation, and expressed by cells presenting antigenic peptides to CD4^+^ T cells (antigen-presenting cells). Indeed, while positivity for HLA-DPA1 or DQB1*0603 is thought to have a protective effect, DQB1*0602 (‘DQ0602’) is considered a genetic marker that strongly predisposes for NT1 (29, 35). The latter is based on the observation that 95-98% of NT1 patients are positive for this HLA-II allele, and that DQ0602 homozygosity (*versus* heterozygosity) significantly increases susceptibility (30, 35–37), linking this allele tightly to the risk of NT1. However, the relative commonness of DQ0602-positivity, found in 15–30% of the general population, also strongly suggests a role for environmental triggers in the overall NT1 etiology (36, 38).

## Pharmacoepidemiological evidence

### Safety signal with Pandemrix

The first reports of narcolepsy in children and adolescents vaccinated with *Pandemrix* emerged in 2010 in Finland and Sweden, the ‘signaling countries’ (1, 39). The timeline and key events are summarized in a publication by M. Sturkenboom (39): “*a suspicion of the association between Pandemrix^®^ and narcolepsy in a child had already been noticed by Dr. Partinen in December 2009 and this association was discussed among neurologists in February 2010 in Finland*”, and: “*a safety signal around Pandemrix, an AS03 adjuvanted influenza A(H1N1) pdm09 vaccine potentially causing narcolepsy in children and adolescents became public in August 2010, long after cessation of the influenza A(H1N1) pdm09 campaigns in Europe*”. Across Europe, the accumulating reports prompted several single-country, retrospective pharmacoepidemiological studies, as well as an extensive multinational case-control study by the ‘VAESCO’ consortium, which confirmed an association for 5–19-year-olds in the signaling countries (1–4, 39, 40). Subsequent European studies estimated relative risks (95% confidence intervals) ranging from 1.5–25.0 (0.3–48.5) for children/adolescents and 1.1–18.8 (0.6–207.4) for adults (2, 8), and attributable risks (excess cases/100,000 vaccinees) of 1.4–8 and 1.0, respectively (1, 2).

### Other adjuvanted H1N1 vaccines

A specific study was launched in Quebec (Canada), to investigate whether any potential association would exist between *Arepanrix* and narcolepsy, based on validated cases of narcolepsy from sleep centers (41). In the primary analysis of this study (41), the relative risk was estimated at 4.3 (95% confidence interval: 1.5–11.1). This estimate is not incompatible with the range of the relative risks observed for *Pandemrix* (1). The authors of the Quebec study concluded by stating that their results were consistent with a risk of narcolepsy following administration of *Arepanrix*, but that the attributable risk was of small magnitude (approximately one case per million) (41). Attributable risks for *Pandemrix* were around 1 per 20,000 (1). Furthermore, secondary analyses of the Quebec study, intended to address the biases differently, have produced lower (and non-significant) relative risks (8, 41). Such a large heterogeneity in relative risk estimates between different analyses of the same study were also typical of the studies on *Pandemrix*.

Other measures of relative risk were produced for Canada in the SOMNIA study (3), a global retrospective and observational study with data from three provinces (Ontario, Manitoba, and Alberta), of which the case-control study in Ontario did not detect any increased risk. In all three provinces, the overall incidence rate of narcolepsy was not higher after *versus* before the vaccination campaign. It should also be noted that this study only detected such an increase in Sweden (the ‘signaling’ country for narcolepsy) but not in any of the other six included countries. In addition, no signal was observed in this study for the *Focetria* pandemic H1N1 vaccine (Novartis; adjuvanted with MF59 (7), another oil-in-water emulsion), of which 12 million doses were administered in Europe, to 8 million individuals across all ages (5, 42). Finally, it was concluded in the SOMNIA study that no changes in narcolepsy incidence were observed between the period after the initiation of the adjuvanted A/H1N1pdm09 vaccination and the period before virus circulation, in any age group or country (3), except for two countries where an increased risk was observed: Sweden, one of the signaling countries, and Taiwan, where the incidence increased concomitantly with influenza virus circulation, in the absence of *Pandemrix* or *Arepanrix* vaccination (3, 43).

In summary, the associations between *Pandemrix* or *Arepanrix* with narcolepsy were measured in different regions. The heterogeneity of relative risk estimates between the two vaccines is not necessarily larger than the heterogeneity of the estimates between the different, more numerous studies performed for *Pandemrix*.

### Role of natural infection

A putative role of natural infection was initially postulated by Han et al. in 2011, based on data from Beijing, China (44). Epidemiological studies of the Chinese Narcolepsy Cohort identified a seasonal increase in narcolepsy onset during the pandemic—in a context of very low vaccine coverage and no vaccination with *Pandemrix*—followed by a decrease in the two years after the pandemic (45). Furthermore, Huang et al. reported an increased narcolepsy incidence in Taiwan during the pandemic that was not associated with pandemic vaccines, which in this region were either non-adjuvanted or MF59-adjuvanted vaccines (43). An etiological role for influenza infection may also explain the chronology of NT1 incidence seen in Germany in the same period (46). Finally, whereas the Quebec data did not suggest a strong evidence of a risk associated with *Arepanrix* of a similar magnitude as that with *Pandemrix*, the local case numbers did increase slightly during the first (spring) pandemic wave in the absence of vaccination (41). Overall, this body of evidence supports a putative role for natural infection as a risk factor for NT1. This is aligned with the outcomes of multiple systematic reviews, meta-analyses and multi-bias modelling studies (1, 2, 4, 39–41, 47). Across these studies and other communications (48), the various identified confounders (e.g., faster diagnoses in individuals exposed *versus* non-exposed to *Pandemrix*, geographical differences in the H1N1 epidemic curve) consistently included the presence of infection as a risk factor.

It has long been recognized that infectious pathogens can trigger or exacerbate autoimmune diseases [reviewed in (49)], and for narcolepsy with cataplexy, a link with *Streptococcus* infection has been reported (50–52). Narcolepsy etiology could also be linked to natural A/H1N1pdm09 infection (5, 25, 50), as supported by several lines of published evidence. First, both influenza infection and unexplained fevers were identified as increasing the risk of narcolepsy in a case-control study (53). Further evidence is provided by the up to four-fold increase (compared with other years) in cases in 2010 in the abovementioned Chinese data from Han *et al*, in the absence of vaccination (44, 45, 54). More recently, a modelling study of *de novo* European cases concluded that the NT1 peak in children detected in 2013 (thus in the absence of recent *Pandemrix* vaccination), was most likely triggered by other risk factors, such as viral infections caused by H1N1 recirculation, or circulation of other/new influenza strains, e.g., influenza B (55). A recent study by Stowe et al. shows that an increased risk of NT1 associated with *Pandemrix* vaccination was “confined to those with onset within the first 12 months with a return to baseline thereafter” (56). This limited (~1 year) timeframe in which the vaccine could be linked to NT1 onsets thus suggests that thereafter other risk factors would have become more likely causes.

Finally, the likelihood of an infectious etiology aligns with the relative chronology of pandemic waves and rises in narcolepsy with cataplexy cases seen globally (41, 43, 46), as exemplified by the Quebec Spring wave in 2009. In several European countries, the pandemic peak preceded the maximum capacity of the vaccination campaigns, with only a short duration between these events (2). For example, mathematical modelling using real-world evidence suggested that most (66%) Norwegian children aged 10-20 years had been asymptomatically or symptomatically exposed to the virus before vaccination (though separating peaks in background illness from vaccine-induced effects proved difficult) (2, 57). A role of preceding A/H1N1pdm09 infection in narcolepsy etiology was however called into question in a serological survey of Finnish patients (58), though the assay characteristics and data interpretation were queried (59). Nonetheless, the overall evidence indicates that a role for A/H1N1pdm09 infection cannot be ruled out, and that infection may act as a confounder in the apparent association between vaccination and NT1. This points again towards a potential role for influenza antigen(s) impacting the interpretation of patient data, both by complicating the attribution of cases to either the vaccine or the infection, and by generating bias (2, 57). Indeed, the vaccinated population, which may have been exposed to the virus and/or had symptoms before vaccination, was also more prone to seek care and get diagnosed as a result of the signal, due to widespread media and public health attention (2, 57).

The strong link of this rare immunopathologic disorder with a single predisposing HLA-II allele, combined with the increase in NT1 incidence in non-vaccinated populations during A/H1N1pdm09 circulation (44–46, 54), pointed towards a multifactorial yet highly specific disease etiology, in which T cells (rather than potential autoantibodies) take center stage. This key assumption informed the mechanistic research plan, performed as part of GSK’s commitment to the European Medicines Agency, to further investigate the signal (9). Interestingly, an extensive 2014 dataset spanning most of the Swedish population (61%; ~6 million persons including 3.3 million *Pandemrix* vaccinees), showed that apart from NT1, no neurological or autoimmune disease was reported at an increased incidence (60). This observation supported the initial assumption on the specificity of the etiology of the NT1 cases reported after the pandemic.

## Clinical and translational mechanistic research

### The T-cell etiology hypothesis

The tight association of NT1 with the HLA-II DQ0602 allele and its function in CD4^+^ T-cell antigen presentation, suggested a disease mechanism involving CD4^+^ T cells with T-cell receptors (TCRs) specifically recognizing peptides from HCRT-secreting neurons, bound to the endogenous DQ0602 allele. The essential role of HCRT deficiency in the etiology of NT1 led to the assumption that this signature protein of HCRT neurons itself could be the autoantigen. HCRT neurons would then act as the prime autoimmune targets for direct and/or cytokine-mediated attacks by CD4^+^ or CD8^+^ T cells. A putative T-cell-mediated etiology of NT1 was further supported by several NT1-associated gene signatures identified in the last decade, which, jointly with DQ0602, formed the basis of the genetic predisposition. These findings included a single nucleotide polymorphism (SNP) in a gene segment of the TCR’s α-chain (TRAJ24) serving as a predisposing marker, and other mutations in immune-regulating genes (e.g. *CTSH*, *P2RY11* and *IFNAR1*) which are thought to affect T-cell activation (35, 61–63).

### Immunological research to evaluate the CD4^+^ T-cell etiology hypothesis

**Figure 1:**
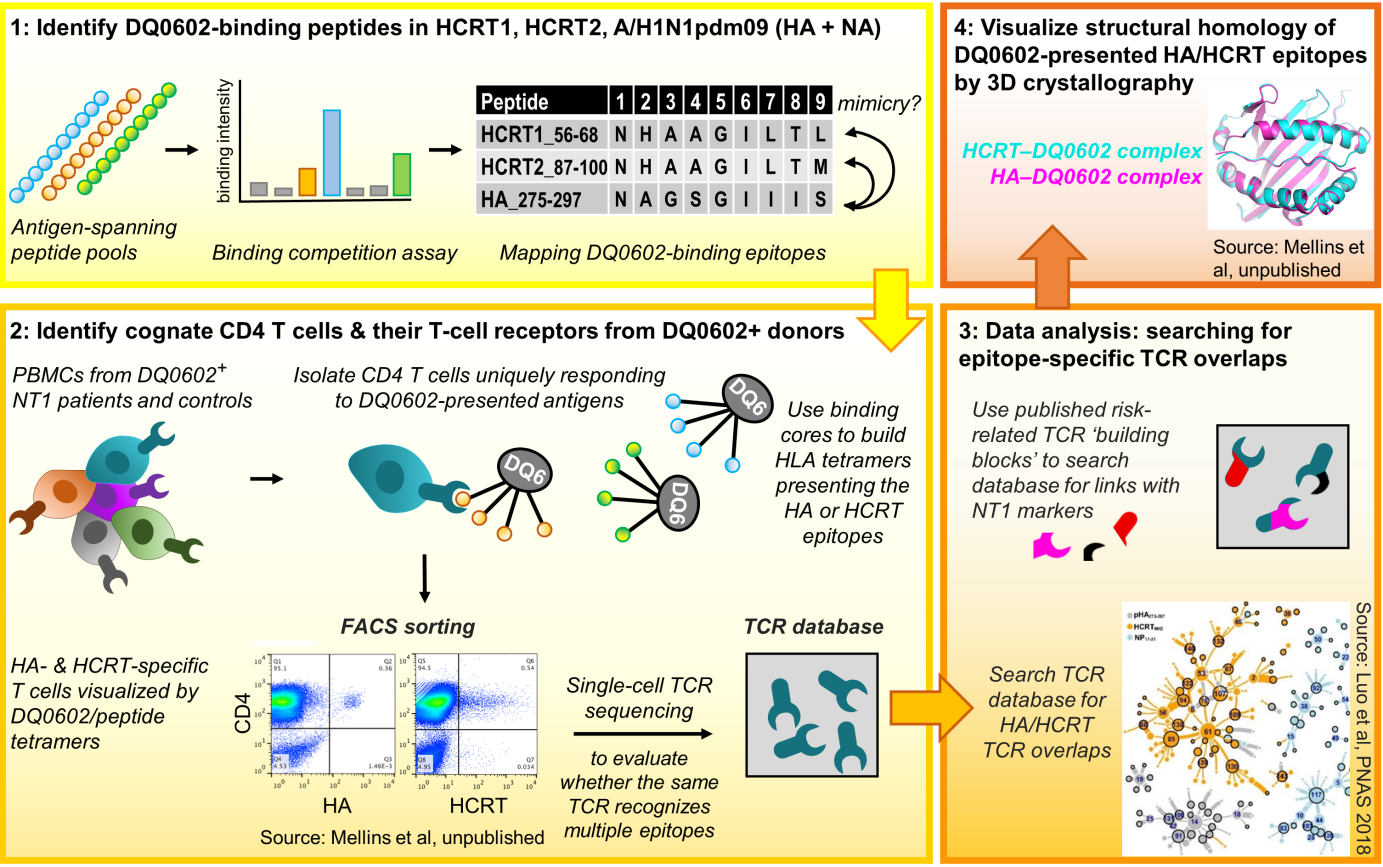
Research workflow [see refs (31, 64) for details]. Figure represents the four workflow steps and tenets. Step 1: Pools of overlapping 15-mer peptides spanning the A/H1N1pdm09 haemagglutinin (HA)/neuraminidase (NA), hypocretin (HCRT)1 and HCRT2 sequences were used to measure binding to DQB1*0602 (DQ0602). Then, the peptides’ uniqueness for A/H1N1pdm09 and putative similarities between the HA and HCRT sequences were determined, with peptide sequence homology considered a first indication for putative molecular mimicry. Step 2: using DQ0602-positive donor CD4^+^ T cells and the human leukocyte antigen class II (HLA-II) tetramers, the peptides from Step 1 were assayed for their abilities to promote T-cell activation and recognition. Isolated tetramer-binding cells were subjected to single-cell T-cell receptor (TCR) sequencing. Step 3: TCR databases were inspected for cross-reactivity and the presence of type-1 narcolepsy (NT1)-associated mutations. The inset [from ref (31)] shows the clustering and overlap of TCR sequences found using tetramers with peptides from nucleoprotein control (NP; blue), HCRT (orange), or HA (grey), allowing to identify cross-reactive TCR families. In Step 4, X-ray diffraction and crystallography analyses were used to investigate structural/conformational similarities between the peptides bound to the DQ0602 groove, to complement the sequential homology data from Step 1. PBMCs, peripheral blood mononuclear cells. FACS, fluorescence-activated cell sorting. Data are shown for illustrative purposes only.

Several publications that emerged over the last decade have begun to shed light on the potential role of T-cell responses in the context of NT1 (31, 64–71). These studies demonstrated amongst others that HCRT can be a target for both CD4^+^ and CD8^+^ T cells, and provided some indications for cross-reactivity. An overview of the mechanistic insights from this research on NT1-related T-cell immunology, which was performed by multiple research groups (31, 64–71), is discussed below. The seminal data, identifying HCRT as a potential T-cell target in NT1, were published by Latorre and coworkers in 2018 (70). These data led several authors to propose a role of autoimmune T cells in NT1 (36, 72). Investigations into a potential T-cell–mediated etiology for NT1 continued by evaluating potential T-cell cross-reactivity between HCRT and H1N1 influenza proteins. This approach was based on mapping DQ0602-restricted epitopes of the four target proteins (A/H1N1pdm09 HA/NA, HCRT1/2), using overlapping 15-mer peptides spanning their sequences. Thus, the central tenets guiding the identification of potentially cross-reactive CD4^+^ T cells (**Figure 1**) were:

1. to identify DQ0602-restricted A/H1N1pdm09 and HCRT peptides, and assess potential sequence homology,
2. to visualize CD4^+^ T cells recognizing these epitopes, and use their TCR sequences as unique identifiers to search for potential HA/HCRT cross-reactivity,
3. to analyze the resulting single-cell TCR sequence databases, with a specific interest in the known NT1-associated SNP,
4. to assess the peptides’ conformational homology when bound to the DQ0602 groove, to complement the sequential homology data from step 1, and confirm that molecular mimicry (i.e., sequence or structural similarities shared between the viral antigens and self-antigen(s) as T-cell targets), underpins their recognition by cross-reactive T cells.

The binding to DQ0602 of the individual peptides was measured, and the binding peptides were then inspected for their uniqueness to the H1N1 virus and for putative HA/HCRT sequence similarities (31, 64–67).

Next (*step 2* above), the peptides were assayed for T-cell activation/recognition using DQ0602-positive donor CD4^+^ T cells. To ensure DQ0602 specificity, two approaches were followed: stimulating purified CD4^+^ T cells using antigen-presenting cells expressing solely DQ0602 (9, 31), or, employing HLAII-DQ0602–peptide tetramers to directly visualize/isolate cognate CD4^+^ T cells (31, 64). While this initially allowed the detection of CD4^+^ T cells with some evidence of cross-reactivity (9), refinement was achieved by subjecting isolated CD4^+^ T cells to single-cell TCR sequence analysis, a technology first reported in 2014 (73). This allowed researchers to directly compare the TCRs recognizing HA or HCRT epitopes, and to define cross-reactivity as TCR sequence identity. *In silico* interrogation of the TCR-sequence databases for HA/HCRT epitope homologies (*step 3*) led to the identification of TCR-identical, DQ0602-restricted CD4^+^ T cells isolated with the HA and HCRT tetramers. These cells were detected in most of the investigated patients with NT1, but, also in around half of the DQ0602-matched controls (9, 31). Given the predisposing role of the TRAJ24 SNP (35, 62), the presence/absence of this ‘risk’ allele received particular attention. The mutation in TRAJ24 was identified in two patient cases and in one control (31), and matched the phenotype of a patient TCR (TCR27) carrying this mutation (64). Collectively, this work identified HCRT peptides as likely T-cell targets and autoantigens, but importantly, only in their bioactive amidated form (31). The short half-times of amidated peptides could then explain why T cells recognizing these peptides might escape tolerance induction in the thymus. Whereas these data provide interesting insights pointing towards T-cell cross-reactivity, it was clear that more research was needed.

Detection of rare and/or cross-reactive T cells was facilitated by combining DQ0602-tetramers with single-cell TCR sequencing. Single-cell analysis was important because bulk stimulation assays using the same peptides failed to detect cross-reactivity on the basis of TCR sequence analysis (67). The latter was likely due to a low frequency of cross-reactive T cells in the pool of cognate cells (69). Nevertheless, a potential role of cross-reactivity for DQ0602-restricted epitopes was further supported by a study comparing HCRT-specific T-cell responses between pediatric patients with NT1 and healthy control children, using peptide stimulation and intracellular cytokine staining (68). This analysis revealed that the NT1 patients displayed higher HCRT-specific CD4^+^/CD8^+^ T-cell frequencies, which were amplified by priming with influenza virus peptides. Higher HCRT-specific CD8^+^ T-cell responses in patients *versus* DQ0602-matched controls were also found by Pedersen et al. (71), using DNA barcode-labeled HLA-multimers to track the antigen specificities of the investigated T cells.

Finally, the notion of peptide mimicry was supported by studying three-dimensional (3D) structures of the HA and HCRT peptides in the DQ0602 groove, using X-ray crystallography (*step 4*). Structural analysis of the crystals synthesized of DQ0602 complexes with HA or HCRT peptides in their binding grooves, revealed similarities that were not obvious from the sequences (64). Indeed, whereas the HA_275-287_, HCRT_56-68_ (HCRT1) and HCRT_87-99_ (HCRT2) sequences displayed differences in five of their nine core residues, conformational homologies were more remarkable at the 3D-level (67). Nevertheless and in apparent contrast, Latorre et al. identified HCRT-specific CD4^+^ and CD8^+^ T cells which did not display any cross-reactivity with influenza antigens (70). Using different detection methods, these authors found that the HCRT-specific CD4^+^ T cells were HLA-DR–restricted, as well as undetectable in healthy DQ0602-positive controls and thus disease-specific.

Collectively, the T-cell data are consistent with a model in which a DQ0602-restricted and cross-reactive CD4^+^ T-cell response may act as the *initiator* of the autoimmune response. This implies that phenotypic differences between patients and controls may be important, and that cross-reactive CD4^+^ T cells *per se* do not represent a biomarker for disease. Hence, the critical event would likely be the loss of peripheral tolerance mechanisms, leading to the presence of unchecked activated autoreactive CD4^+^ T cells in the brain. It was hypothesized that, subsequently, local immune activation in the hypothalamus would (due to a phenomenon known as ‘epitope spreading’) lead to involvement of other T cells with different targets, including HLA-DR–restricted CD4^+^ and CD8^+^ T cells (36). Because these responses would be activated *after* disease initiation by cross-reactive T cells, it is expected that such secondary immune responses would then be more disease-specific and not necessarily cross-reactive. This would be supported by the peak response of cells with the DQ0602-based specificity detected at onset of the disease (31). Finally, involvement of HCRT-specific CD8^+^ T cells in the response may provide an explanation for how exactly CD4^+^ T cells could orchestrate an autoimmune response resulting in the loss of HCRT neurons, given that these neurons do not express HLA-II.

In summary, rare cross-reactive DQ0602-restricted CD4^+^ T cells could play a pivotal role as a trigger in disease onset under specific conditions, most likely involving local inflammation facilitating access across the blood-brain barrier. Once initiated, other cells, such as CD8^+^ and CD4^+^ T cells with different HLA restrictions and specificities, could become involved. It then follows that there would be neither an immunological need, nor a plausible mechanism, for these cells to be cross-reactive.

### Extending the CD4^+^ T-cell etiology hypothesis: non-clinical data

The human data generated so far provided potential evidence for the T-cell recognition of HCRT, as well as indications for cross-reactivity with HA, supporting a plausible immunological mechanism underlying the pathogenicity. Still, they do not connect the peripheral infectious events with the hypothetical local autoimmune responses in the human brain. Specifically, insight was needed into which CNS events would be required to first activate peripheral HA/HCRT-cross-reactive CD4^+^ T cells to cross the blood-brain barrier, and then, instigate the targeting of HCRT neurons in the CNS. This was where animal models came into play. However, the models created since the discovery of HCRT and HCRT-Rs, such as mice deficient for these components or narcoleptic dog models, did not fit the purpose because they do not allow to study disease etiology. To address this, novel mouse models were generated, as reported by two independent teams in 2016 (74, 75).

Transgenic mice artificially expressing HA by HCRT neurons under control of the HCRT promoter (thus bypassing the need for peptide mimicry, since it forces expression of an influenza epitope in the CNS) were used by Bernard-Valnet *et al*, to demonstrate a role for HA-specific T cells in the immune attack of HCRT neurons (74). While hypothalamic inflammation was seen following injection of HA-specific CD4^+^ and/or CD8^+^ T cells, narcolepsy-like symptoms were only observed when activated CD8^+^ T cells were administered to the mice, pointing towards a role for CD8^+^ T-cell–mediated cytotoxicity. The researchers then went on to evaluate the role of *Pandemrix* vaccination in this model, and their results confirm the data from the original work: induction of HA-specific CD4^+^ and CD8^+^ T cells in the HA-transgenic mouse model led to an “immunopathological process mimicking narcolepsy” (76). Whereas this mouse model was not designed to study T-cell cross-reactivity, the results support a role for self-recognizing CD4^+^ and CD8^+^ T cells in the pathology of NT1. The key role of CD8^+^ T cells in the mouse model is consistent with the abovementioned discovery of HCRT-specific CD8^+^ T cells (70, 71), and with the expectation that CD4^+^ T cells do not kill HCRT neurons, which only express HLA-I and therefore would be recognized by CD8^+^ rather than CD4^+^ T cells. Given the pathogenic role of CD4^+^ T cells suggested by the human data, possibly CD8^+^ T cells are only engaged once an immune response is triggered by cross-reactive CD4^+^ T cells. However, it should be noted that vaccine evaluations have failed to detect the induction of any CD8^+^ T-cells in the blood of human recipients of AS03-adjuvanted vaccines (11, 77, 78).

Tesoriero *et al*, on the other hand, used *Recombinant activating gene 1* (*RAG1*)-knockout (T-cell–lacking) mice to demonstrate that intranasal infection with a neuro-adapted A/H1N1 influenza strain can trigger hypothalamic neuronal infection (75). In these mice, the virus travelled *via* the trigeminal nerve through the blood-brain barrier into the hypothalamus where it infected HCRT neurons, which in turn triggered NT1-like symptoms. While it has been argued that this mechanism supports a virus-based rather than a T-cell–mediated auto-immune etiology (79), both are not necessarily mutually exclusive, because infection would serve as a strong T-cell attractant, as explained above.

The model that emerged from the combined murine data explained that (i) local brain inflammation is plausible and can be localized to HCRT-secreting neurons—an event that is likely to attract a T-cell response that originated at the vaccine injection site, infected airways and/or draining lymph nodes, into the brain; and that (ii), if mimicry exists [as modeled by HA in the mouse study (74)], the involved T cells can instigate NT1-like symptoms. However, cotton rat data have indicated that a trigger for such T-cell migration is unlikely to come from an intramuscular adjuvanted vaccine alone. Indeed, repeated injections of either AS03-adjuvanted vaccine or AS03 alone did not cause any inflammation in the CNS (80). Moreover, no changes in the blood-brain barrier integrity were observed in these animals (80). The collective animal data then informed a second hypothesis refining the mechanistic model, and confirmed/extended the earlier suggested necessity of an environmental trigger preceding or acting in parallel with the vaccination (5, 81).

### Connecting the dots: the “two-hit” NT1 etiology hypothesis

Based on the collective immunological and epidemiological evidence, a hypothetical mechanistic model is proposed that connects CD4^+^ T-cells targeting HCRT peptide sequences, with T cells recognizing putative cross-reactive epitopes in H1N1 antigens, such as the HA and NA antigens, given that these two antigens were present in both the H1N1 virus and the vaccine. This model was based on assumed sequence or structural similarities shared between the viral antigens and self-antigen(s) as T-cell targets (called ‘molecular mimicry’), such that CD4^+^ T cells responding to the influenza HA peptides could conceivably cross-react with peptide sequences from HCRT (9, 31). The projected sequence of events would then be that secreted HCRT peptides would be picked up by antigen-presenting cells surrounding the HCRT neurons, such as microglia, making HCRT ‘visible’ to cognate CD4^+^ T cells. Consequently, in a predisposed host, these HLA-II–positive cells expressing DQ0602, would then present DQ0602-binding peptides to CD4^+^ T cells. In parallel, peptides from influenza antigens, such as those in the HA protein, are presented to influenza-specific CD4^+^ T cells in peripheral lymph nodes following infection and/or vaccination. Such ‘mimicry peptides’ can then be recognized by HA/HCRT cross-reactive CD4^+^ T cells. Any inflammation in the brain, caused by, for example, infection or other trauma, could entice these T cells to cross the blood-brain barrier, in order to survey the local inflammation. These activated cross-reactive CD4^+^ T cells could then recognize the mimicry HCRT peptide presented by microglia, and trigger autoimmunity (36).

To explain the high specificity of the peptide-DQ0602 binding, it was also assumed that the mimicry peptide, when presented by DQ0602-expressing microglia, displays a unique conformation. In this model, a slightly different way of binding of the same peptide to, for example, a protective allele such as DQ0603 (35), would prevent cross-reactive T cells from becoming activated, because the peptide appears different from the perspective of the TCR.

**Figure 2:**
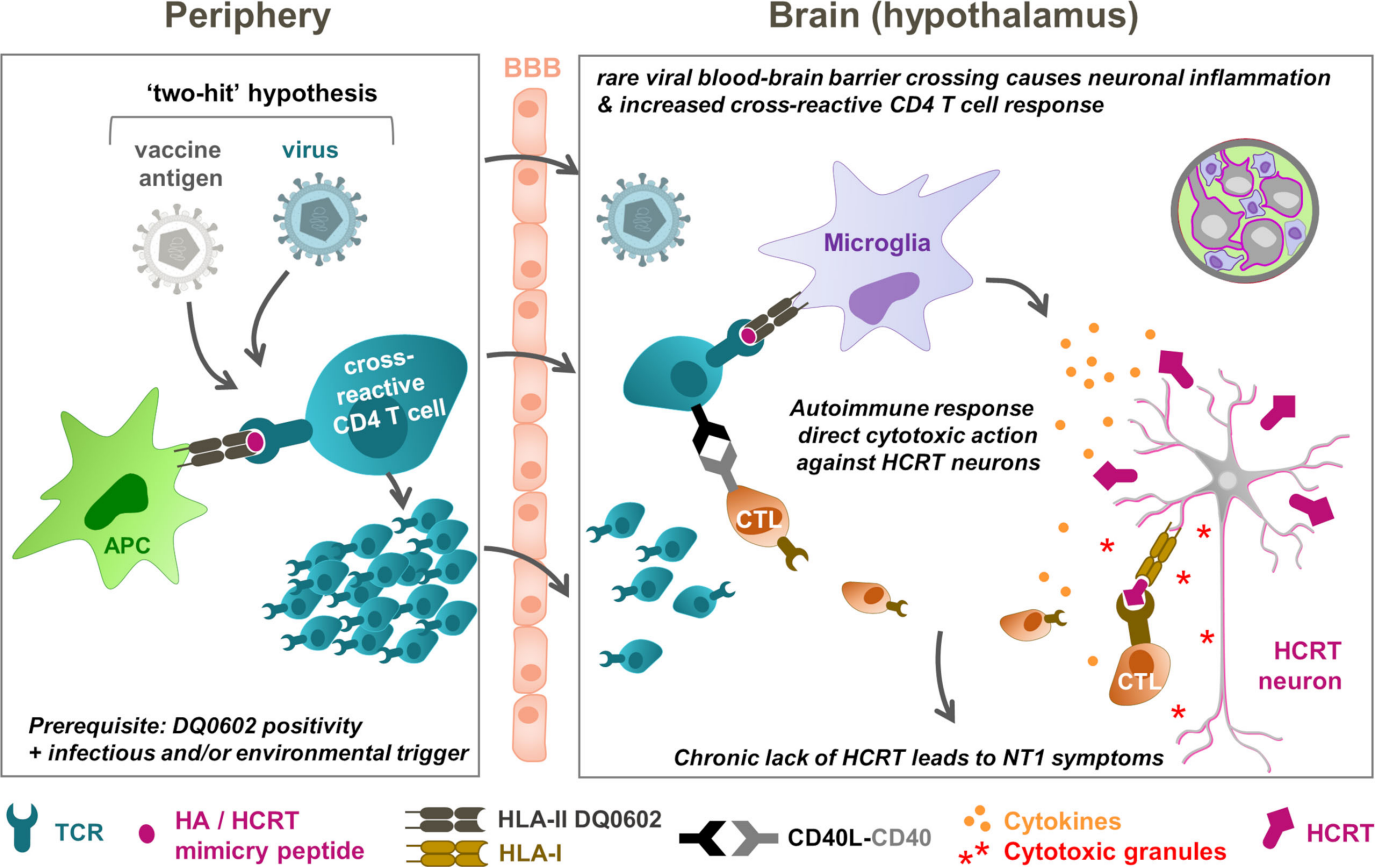
NT1 immunopathogenesis model: T-cell cross-reactivity and “two-hit” hypotheses. The hypothetical model connects peripheral (left) and hypothalamic (right) events. In this model, rare conditions, including brain injuries or pathogens (A/H1N1pdm09 virus, *Streptococcus*) crossing the blood-brain barrier (BBB), prime for local brain inflammation and, due to immune surveillance, the expansion and migration of T cells from the periphery into the brain. In DQ0602-positive persons, these T-cell responses could include DQB1*0602 (DQ0602)-restricted, haemagglutinin (HA)/hypocretin (HCRT)–cross-reactive CD4^+^ T cells, as a “first hit”. These low-frequency immune responses could then be amplified by an independent event (“second hit”), which in highly rare occasions might have been a vaccination that stimulates CD4^+^ T-cell responses. Further response amplification due to ‘epitope spreading’ and autoimmune recognition of epitopes in HCRT peptides from the hypothalamic HCRT neurons, may then activate the HCRT-specific T cells to launch immune attacks on these neurons. These attacks are mediated by cytokine release and cytotoxic (CD8^+^) T lymphocyte (CTLs) that are ‘licensed to kill’ by the CD4^+^ T cells. The subsequently released cytotoxic granules, containing perforin and granzymes, directly attack the virus-infected neurons upon HLA-I mediated antigen recognition. The progressive loss of the HCRT neurons ultimately results in type-1 narcolepsy (NT1) symptoms in the affected person. Note that failure of peripheral tolerance may constitute a third parameter that could affect risk. APC, antigen-presenting cell. TCR, T-cell receptor. HLA, human leukocyte antigen. CD40L, CD40 ligand.

The available evidence demonstrates that HA/HCRT peptide similarity can occur independently from NT1 symptoms, as both healthy subjects and patients displayed CD4^+^ T cells recognizing the presented DQ0602-HA/HCRT peptide complexes (31). It follows therefore that these cells may be required but are, by themselves, insufficient to cause narcolepsy (NT1) symptoms. Several relatively rare conditions can independently trigger local CNS inflammation, whereas the existence of a regulatory self-reactive immune network in the CNS should also be considered (82). The potential inflammation triggers include brain injuries, or the crossing of pathogens (e.g., A/H1N1pdm09, *Streptococcus*) through the blood-brain barrier during infection, but likely not the adjuvanted A/H1N1pdm09 vaccination itself (80). Then, due to natural immune surveillance, the local inflammation could result in T-cell expansion and migration from the periphery into the brain. Importantly, in some DQ0602-positive predisposed individuals, such a response could include the rare DQ0602-restricted HA/HCRT cross-reactive CD4^+^ T cells (“first hit”), which could then be amplified by an independent event at the time of, or soon after infection (“second hit”; **Figure 2**) (5, 81). It is tempting to speculate that the existence of an immune regulatory network in the CNS (82) could necessitate a third hit, i.e., failure of the immune tolerance mechanism. We hypothesize, based on the accumulated evidence, that in a very small proportion of predisposed individuals, vaccination may have amplified the already triggered immune cascade, because administration of the adjuvanted vaccines has been shown to stimulate CD4^+^ (but not CD8^+^) T-cell responses (11, 77, 78). Due to the combined action of both factors, and supported by the hypothesis of epitope spreading (36), local autoimmune recognition of epitopes from hypothalamic HCRT neurons may then trigger the activation of HCRT-specific CD4^+^ and CD8^+^ T cells. This might lead to the progressive loss of HCRT neurons, and, ultimately, to NT1 symptoms in the affected individuals.

In summary, if the A/H1N1pdm09 viral preexposure suggested by some pharmaco-epidemiological studies is confirmed, a plausible hypothesis is that the ‘priming’ of the cross-reactive CD4^+^ T-cell response by natural infection was further activated following pandemic influenza vaccination. In addition, if, as hypothesized, the putative mimicry peptide is indeed specific to A/H1N1pdm09 protein, this would explain several epidemiological observations demonstrating the association between an increased risk of NT1 and A/H1N1pdm09 infection, including (i) the absence of a risk increase prior to 2009, when the mimicry peptide was absent; (ii) the 2010 peak in China detected in the absence of *Pandemrix* vaccination (44, 45, 54); and, possibly, (iii) the European incidence peak observed in 2013 (55).

### Alternative hypotheses

Besides the T-cell hypothesis, several other possible scientific explanations based on *in silico* or *in vivo* data have been proposed, guided by purported differences between *Pandemrix*, *Arepanrix* and *Focetria*. **Table 1** presents an overview of these studies and their limitations, in several cases pertaining to the absence of plausible mechanistic links and/or confirming data (32, 84, 85, 88). The scientific arguments centered mostly on potential variations in the HA or nucleoprotein (NP) vaccine antigens (86, 87, 89, 92, 94) rather than on adjuvant differences, which led the European Medicines Agency to conclude that “*based on the evidence generated so far, a hypothesis that takes into account the potential role of antigen is more likely to explain the increased risk of narcolepsy observed with Pandemrix than hypotheses that are based on a direct role for the AS03 adjuvant*” (9). The latter (adjuvant-based) hypotheses, focusing on α-tocopherol (contained only in AS03) were the topic of only one report (83).

**Table 1:** Alternative hypotheses.

Focus	Observations and Hypotheses	Counter argument
α-tocopherol in adjuvant	Neuronal cells exposed *in vitro* to free α-tocopherol displayed increased HCRT expression, presumably leading to higher neuron sensitivity to apoptosis (83)	• Different behavior α-tocopherol *in vivo* vs *in vitro* (84) • No migration of formulated α-tocopherol to CNS (85)
Change in HA of vaccine Ag	The increased frequency of a single amino-acid change in *Pandemrix* and wild-type virus compared to *Arepanrix* or other H1N1 vaccines was hypothesized to have altered the predisposition to NT1, by changing the binding to DQ0602 (86)	No mechanistic link between reduced binding to DQ0602 and NT1 pathogenesis
Change in NP of vaccine Ag enhances cross-reactive Ab responses	Polysorbate detergent used in *Pandemrix* production allegedly induces structural changes in the NP of the vaccine Ag, which increases anti-NP Ab responses cross-reacting with DQ0602 (87)	• *Pandemrix* and *Arepanrix* NP sequences originate from PR8 strain rather than from A/H1N1pdm09 (88) • No mechanistic link between anti-NP Abs and NT1 development
	Alleged similarity in NP and HCRT-R2 sequences (dubbed the “mimicry sequence”) is a target of cross-reactive anti-NP/HCRT-R2 Ab responses, which were claimed to be more abundant in vaccinees of *Pandemrix vs Focetria* due to the relative enrichment of NP in *Pandemrix*. This would link *Pandemrix* vaccination with increased NT1 risk (89)	• No clear mechanistic involvement of such cross-reactive Abs in the loss of HCRT neurons (88): – HCRT neurons do not express HCRT-R2 – No need for DQ0602 binding of “mimicry peptide” to induce cross-reactive anti-HCRT-R2 Abs (32) • Mimicry sequence not confirmed by independent analyses or GenBank database (entry KJ_942731) (88) • Cross-reactive Abs were detected in vaccinees both with and without NT1 symptoms (89) • No cross-reactive Abs detected in later studies (31, 90, 91)
Vaccine Ag target of Abs against ganglioside GM3	Anti-GM3 auto-Abs found more often in vaccinated *vs* unvaccinated persons (18% *vs* 7%; P=0.035), and in *Pandemrix*-vaccinated NT1 patients *vs* healthy controls (15% *vs* 4%; *p* = 0.047). This led to the claims that anti-GM3 Ab responses are associated with DQ0602 (*p* = 0.016) in both vaccinated patients and controls, collectively linking *Pandemrix* to NT1 risk (92)	• Key results are borderline statistically significant (92) • The relationship, if any, between the Ab responses and any A/H1N1pdm09 protein is unclear • The hypothesis was discarded in earlier research (93)
Other auto-Ab responses	Using microarray screening technology, other Ab targets were found to be recognized differently in NT1 patients and healthy controls (94)	• Unclear relationship, if any, between these proteins and any A/H1N1pdm09 proteins • Unclear if the proteins are indeed autoimmune targets

Ag, antigen; Ab, antibody; CNS, central nervous system; HA, haemagglutinin; HCRT-R2, hypocretin receptor type 2; NP, nucleoprotein; NT1, narcolepsy type I; DQ0602, DQB1*0602; A/H1N1pdm09, A/California/7/2009 H1N1.

Rather than a T-cell–based etiology, several of the studies proposing alternative models suggested a central role for antibodies, including cross-reactive anti-HCRT-R2/NP antibodies, antibodies against NP itself, and anti-ganglioside or other autoantibodies (87, 89, 92, 94). In particular, the hypothesized causal role of NP/HCRT-R2-cross-reactive antibodies has been challenged in later studies, which all failed to reproduce these cross-reactive antibody profiles (31, 90, 91, 93). It is therefore not straightforward to translate the collective antibody data into a plausible mechanistic model (94). Furthermore, recent data by Lind et al. suggested that HA antibody responses are both qualitatively and quantitatively comparable between NT1 cases and controls, leading these authors to conclude that HA antibodies are likely not involved in the pathogenesis (95).

Overall, the field has moved to a stronger emphasis on a mechanistic role for T cells in the etiology of NT1, as exemplified by recent clinical data (96). Confirmation of the T-cell cross-reactivity was not only provided by the mechanistic murine data (74, 75) (§ Extending the CD4+ T-cell etiology hypothesis: non-clinical data), but also by a recent publication postulating mimicry between a DQ0602-restricted epitope in A/H1N1pdm09 NA and a self-epitope from a protein (protein-O-mannosyl-transferase; POMT1) expressed in the CNS (97). The authors concluded that the data identified POMT1 as a potential autoantigen for B and T cells, though it has no known links to NT1.

## Discussion

From the in-depth pharmacoepidemiological and immunological studies that were triggered by the increased NT1 incidence in Europe immediately following the A/H1N1pdm09 influenza pandemic, a model has gradually emerged which plausibly explains the sequential immunological events underpinning the increased risk. In brief, the hypothetical mechanism centers on a possible molecular mimicry between antigens in the A/H1N1pdm09 virus and vaccines and in HCRT. The consequence of this mimicry may be to trigger an autoimmune response targeting HCRT-producing neurons, activated by local responses of CD4^+^ T cells recognizing peptides from HCRT. The reaction could be primed by rare pathogen-instigated brain inflammation, or by genetically driven loss of peripheral tolerance, attracting DQB1*0602-restricted, cross-reactive T cells from the periphery. The frequencies of these T cells in the circulation, and the numbers of these cells subsequently migrating to the brain, could then be amplified by a subsequent event, such as vaccination, effectuating a “two-hit” hypothesis. Failure of peripheral tolerance could be considered a third hit.

The collective *in silico*, *in vitro*, and preclinical *in vivo* data from the research efforts—which are still continuing today—have progressively refined the initial hypothetical model of sequential immunological events, and have filled multiple knowledge gaps. The scientific debate over the last decade has also demonstrated the value of combining immunology research (focusing on T-cell cross-reactivity) with the pharmacoepidemiological evidence. Indeed, this combination has offered plausible explanations within the “two-hit” hypothesis mechanistic framework, and the current review aimed to provide an interpretation of the T-cell data that emerged over the last decade, which fits within this framework. Future research, using single-cell analysis and HLA tetramers, could focus on further characterization of HCRT-specific T cells, including their fine-specificities, phenotypes (effector versus regulatory), and cross-reactivity profiles. Further refinement of mouse models, e.g., DQ0602-transgenic mice, to recapitulate antigen-specificity and mimicry of disease development, would also be of value. Understanding the immunological mechanism(s) underlying the observed increased risk of NT1 is important from the perspectives of public health and the patients, and can ultimately inform future research on NT1 and, potentially, on other autoimmune diseases.

Altogether, though no definitive conclusions can be drawn, the hypothesis of possible molecular mimicry remains plausible to explain the increased risk of NT1 observed following infection and/or vaccination against A/H1N1pdm09 pandemic influenza. A better understanding of the narcolepsy disease mechanisms and of the potential role of cross-reactive CD4^+^ T cells has brought novel insights and refinements of the proposed hypothetical model. In this context, several lines of evidence converge on the notion that the presence of such cross-reactive CD4^+^ T cells alone is not a predictor for the narcolepsy pathogenesis.

## Trademarks

Pandemrix and Arepanrix are trademarks owned by or licensed to the GSK group of companies. Focetria is a trademark of Novartis. All these products are now withdrawn from use in the European Union. The marketing authorisation for Pandemrix and Focetria expired on 13 August 2015 and the marketing authorisation for Arepanrix expired on 13 December 2010.

## Author contributions

All authors contributed to the research, reviewed the literature, provided substantial input and reviewed the paper. All authors approved the final article and are accountable for all aspects of the work. For RvdM, it is noted that the views and opinions in this publication are his personal views and opinions.

## Funding

This study received funding from GlaxoSmithKline Biologicals SA, which funded this literature review and took in charge all costs associated with the development and the publication of this manuscript.

## Acknowledgments

The authors thank Fernanda Tavares Da Silva, Vincent Bauchau, Maria de los Angeles Ceregido Perez, Margherita Coccia, Marguerite Koutsoukos, Derek O’Hagan (all GSK) and Norman Begg (independent vaccine consultant) for their critical review of the manuscript. They also thank Ellen Oe (GSK) for providing medical writing support, and Business & Decision Life Sciences platform for editorial assistance and manuscript coordination, on behalf of GSK. Géraldine Drevon (GSK) and Nathalie Arts (Business & Decision Life Sciences) coordinated publication development and editorial support.

## Conflict of interest

SB is employed by the GSK group of companies. RvdM was employed by the GSK group of companies. SB and RvdM hold shares in the GSK group of companies. GlaxoSmithKline Biologicals SA funded this literature review and took in charge all costs associated with the development and the publication of this manuscript.

The funder was involved in the study design, collection, analysis, interpretation of data, the writing of this article and the decision to submit it for publication.

## Publisher’s note

All claims expressed in this article are solely those of the authors and do not necessarily represent those of their affiliated organizations, or those of the publisher, the editors and the reviewers. Any product that may be evaluated in this article, or claim that may be made by its manufacturer, is not guaranteed or endorsed by the publisher.
